# Comparative Evaluation of Silver-Containing Antimicrobial Dressings on In Vitro and In Vivo Processes of Wound Healing

**Published:** 2012-10-11

**Authors:** Matthew E. Hiro, Yvonne N. Pierpont, Francis Ko, Terry E Wright, Martin C. Robson, Wyatt G. Payne

**Affiliations:** ^a^Institute for Tissue Regeneration, Repair, and Rehabilitation, Department of Veterans Affairs, Surgical Service, Bay Pines VA Healthcare System, Bay Pines, Fla.; ^b^Division of Plastic Surgery; ^c^Department of Surgery, University of South Florida, Tampa, Fla.

## Abstract

**Objectives:** To compare the in vitro and in vivo effects of silver products on wound healing. **Methods:** Eight silver products were compared to determine: fibroblast function using fibroblast-populated collagen lattices (FPCLs), fibroblast viability using the Trypan Blue exclusion test, and fibroblast mitochondrial activity using the MTT [yellow tetrazolium salt; 3-(4,5-Dimethylthiazol-2-yl)-2,5-diphenyltetrazolium bromide] assay. In vivo effects of 9 silver products were evaluated utilizing a rat model of contaminated wounds. Serial quantitative bacteriology was performed on tissue biopsies over a 10-day period and serial wound areas were obtained over 12 days. **Results:** Fibroblast cytotoxicity occurred for all of the silver products evaluated. Remaining viable fibroblasts were insufficient to allow FPCL contraction. Mitochondrial activity of the fibroblasts allowed a separation of the various silver compounds. Actisorb Silver and Silvercel had the greatest viable fibroblast activity, but less than the control. Despite in vitro cytotoxicity, all of the silver products except Contreet Foam and Acticoat Moisture Control accelerated wound healing. **Conclusions:** Silver-containing dressings appeared to benefit healing of the wounds. Just as in vitro bacterial analyses do not fully predict the effect of an antimicrobial in the in vivo setting, in vitro cytotoxicity tests do not fully predict the effect of an agent on wound healing trajectories. Because of the varied antimicrobial and wound healing responses among products, a careful consideration of the particular effects of individual silver-containing dressings or drugs is warranted.

Wound healing is the end result of a series of interrelated cellular processes initiated by humoral factors such as cytokine growth factors.^1^ These cellular processes are inhibited by a large tissue bacterial bioburden.^2^ The cytokines and growth factors are also degraded by bacteria.^3^ The level of tissue bacterial bioburden that inhibits healing has been shown in multiple studies to be greater than 1 × 10^5^ or at least 1 × 10^6^ bacteria per gram of tissue.^4^^,^^5^ Such high levels of tissue bacteria can be present without clinical signs of infection, and when present can deleteriously affect wound healing.^6^

Attempts at controlling the tissue bacterial bioburden have been difficult. Systemically administered antibiotics do not effectively decrease the level of bacteria in a chronic granulating wound.^7^ Therefore, topical antimicrobials or temporary biologic dressings have been the methods of choice.^4^^,^^8^ Topical use of antibiotics that are used effectively systemically for purposes other than wound infection is discouraged because of an increased risk for developing allergies or the potential for bacteria to develop resistance to the drug.^9^

Because of the deleterious effect of a high tissue bacterial burden on the processes of wound healing, an effectual antimicrobial agent becomes a therapeutic imperative. Such an agent should be effective as a topical preparation, yet not to be cytotoxic to the cells involved in the wound healing process.^10^ The antibacterial properties of silver have made it an attractive and practical choice for creating silver-based topical creams and dressings to comprise a class of antimicrobial topical treatments that have been used in wound care.^11^^-^13

Topical silver creams and solutions have a broad spectrum of antimicrobial activity, low development of resistance, few adverse reactions, and a low risk of systemic toxicity, but require frequent application, are care-intensive to apply and remove, and are sometimes painful. In contrast, wound dressings containing silver have been introduced in various designs to control the release of silver to the wound allowing the dressings to be changed less frequently.^14^ The authors previously reported on the comparative antibacterial properties of eight silver-containing dressings and 2 nondressing silver agents.^14^

The other issue regarding topical antimicrobial agents and dressings is their potential for cytotoxicity. Fleming has stated that anything that is bactericidal may well be tissuecidal.^10^ Some silver-containing antimicrobials have been found to exert cytotoxic effects on wound tissue and to inhibit keratinocyte production.^15^^-^16 There is also concern that using them on open wounds may be injurious to fibroblasts and inhibit wound healing.^17^^-^19 The purpose of this study was to evaluate the effect on various in vitro and in vivo processes involved in the wound healing using the silver-containing dressings and agents previously reported for their antimicrobial effects.

## METHODS

### In vitro fibroblast function

Fibroblast function was assessed by 3 methods. The first and second methods were the fibroblast-populated collagen lattice (FPCL) and Trypan Blue exclusion assay to assess for functionality and viability. The third was a measure of mitochondrial activity in living fibroblasts using the MTT [yellow tetrazolium salt; 3-(4,5-Dimethylthiazol-2-yl)-2,5-diphenyltetrazolium bromide] assay. The fibroblasts for both tests were prepared by explanation as previously described.^20^ The collagen lattices were prepared from type I rat tail collagen (acetic acid extracted) as recommended by the manufacturer (Upstate Biotechnology, Lake Placid, New York).^20^ Undiluted collagen (1 mL) was placed in 35-mm culture dishes (Falcon 1008) and evenly spread. The dishes were placed in an ammonia vapor chamber for 3 minutes to solidify. Sterile distilled water (5 mL) was added to the culture dishes, allowed to stand for 1 hour, and then aspirated. This was repeated 4 times to remove excess ammonia and the collagen lattices were then incubated for 24 hours at 4°C. Phosphate-buffered saline with 1.0% serum was added to replace the final aspirate. An 18-gauge needle was used to detach the collagen gel lattices from the surface of the culture dishes so that they would be loose and suspended in saline. A total of 45 collagen lattices were prepared to allow triplicate measurement based on eight treatment groups, plus an untreated control. To form the FPCLs, all saline was aspirated from the 35-mm culture dishes containing the lattices. Two mL of 2 × 10^5^ fibroblasts per milliliter were placed on the surface of each of the prefabricated collagen gel lattices.^20^^-^23

Fibroblast-populated collagen lattices were divided into 9 groups. One group was kept as a control with no treatment, and the other 8 groups received one of the 8 types of silver-containing dressings. The silver-containing dressings were laid over the collagen matrix. The 8 types of silver dressings were Acticoat 7 (Smith and Nephew, London, UK), Acticoat Absorbent (Smith and Nephew, London, UK), Acticoat Moisture Control (Smith and Nephew, London, UK), Actisorb (Systagenix, North Yorkshire, UK), Aquacel Ag (ConvaTec Inc, Skillman, New Jersey), Contreet Foam (Coloplast, Minneapolis, Minnesota), Silvercel (Systagenix, North Yorkshire, UK), and Urgotul SSD (silver sulfadiazine) (Laboratoire Urgo, Chenove, France).

The FPCLs were incubated at 37°C in a humidified atmosphere of 5% carbon dioxide. The amount of gel contraction was measured every 24 hours for 5 days.^20^^-^23 Acetate overlays were used for tracing the area of the gels. Gels were performed in triplicate (3 gels) for the fibroblast line established and measurements were then calculated using digital planimetry and Sigma Scan software (Jandel Scientific, Corte Madera, California). Each collagen gel area measurement was converted to reflect percentage of gel contraction.^20^

A one-way analysis of variance was used to determine significant differences among groups. When a difference was identified, a Tukey's test (all pairwise multiple comparisons test) was used to delineate the differences. Sigma Stat statistical software (Jandel Scientific, Corte Madera, California) was used for data analysis.

In addition, the 24-hour FPCLs with silver-containing dressing overlays were examined microscopically and photographed.^24^ One of the gels was evaluated for fibroblast viability at 48 hours utilizing the Trypan Blue exclusion assay. Cell numbers were evaluated spectrophotometrically as a function of mitochondrial activity in living fibroblasts using the MTT assay.^25^ In brief, the MTT assay is as follows: after exposure of 5×10^5^ fibroblasts to the test silver-containing dressings, a 48-hour suspension in Dulbecco's Modified Eagle Medium was reincubated for 2 hours and 4 hours and then exposed to MTT assay. Mitochondrial dehydrogenases from viable fibroblasts cleave the tetrazolium ring, yielding purple formazan crystals. These are dissolved in acidified isopropanol resulting in a purple solution, which can then be spectrophotometrically measured. An increase or decrease in cell numbers results in a concomitant change in the amount of formazan formed, indicating the degree of toxicity caused by the test material.

### In vivo comparative wound model

All procedures followed a protocol approved by the Bay Pines VA Healthcare System Institutional Animal Care and Use Committee. Male Sprague-Dawley rats weighing 275 to 325 g had general anesthesia introduced by intraperitoneal injection of pentobarbital (35 mg/kg). Following satisfactory anesthesia, the animals’ backs were clipped of hair and depilated. Four square 1.5 × 1.5 cm^2^ symmetrical wounds were created in the midline of the back.^26^^-^29 A copper template was used to create a line of 4 square wounds through the skin and panniculus carnosus muscle to the deep fascia of the back. The template was sited centrally in a position that did not allow animals to reach the wounds with their paws or mouths to prevent potential interference with healing from ingestion of the treating agent or from trauma.

The wounds were then treated according to the following protocol: Each of the wounds was inoculated with 5×10^5^ colony forming units (CFUs) of *Escherichia coli* ATCC 25922 (American Type Culture Collection, Rockville, Maryland).^27^^,^^29^ Each animal had the cephalic wound left untreated as a control and the caudad 3 wounds dressed with one of 9 silver-containing dressings.^26^ There were 5 animals (n = 5) in each dressing comparison group (15 wounds). The silver dressings evaluated were Acticoat 7, Acticoat Absorbent, Acticoat Moisture Control, Actisorb, Aquacel Ag, Contreet Foam, Silvercel, Urgotul SSD and Silverlon (Cura Surgical, Geneva, Illinois). In addition, there were 2 control groups, an open control and a closed control treated with Tegaderm (3M, St Paul, Minnesota). Animals were housed in individual cages (following 10 days acclimatization to separate caging) and given food and water ad libitum.

Dressings were changed every 24 hours. Every second day, the animals were reanesthetized and wounds were traced on acetate sheets and biopsied for quantitative bacteriology. Quantitative bacteriology was performed according to the method of Heggers and Robson.^5^ Prior to biopsy, surface swabs were obtained for surface bacterial analyses. The wound surface was then cleansed and biopsies taken with a disposable punch biopsy trephine, and the biopsy weighed aseptically. The specimen was then dipped in alcohol and flamed to remove surface contamination, and then homogenized, after a 1:10 dilution with thioglycollate broth. Serial 10-fold dilutions were prepared and backplated to arrive at an accurate bacterial count.^5^ Animals were followed for 12 days and biopsies obtained on days 2, 4, 6, 8, and 10.

Bacterial results were analyzed by the Duncan's range test (multiple comparisons), the alpha error set at 0.05, and quantitative bacteriology was reported as CFUs per gram of tissue. Analog tracings were made on alternate days on acetate sheets of both the open wound areas and of the advancing full-thickness skin edges of all wounds. To eliminate site-related variability in healing dynamics, the cephalad most wound of each animal was excluded from wound healing data analysis.^30^ Any dried exudate was atraumatically removed prior to any wound tracings or biopsies. Wound areas were measured by digital planimetry of the acetate tracings (Sigma Scan, Jandel Scientific, Corte Madera, California). As described by Hokanson et al,^31^ data acquired from each wound, as well as cumulative data from each group, was plotted graphically. Wound areas were compared at each measurement point using a 1-way analysis of variance, an all pairwise multiple comparisons test (Tukey test), and Mann-Whitney rank sum test to determine significant differences.

## RESULTS

### In vitro fibroblast function

**Figure 1:**
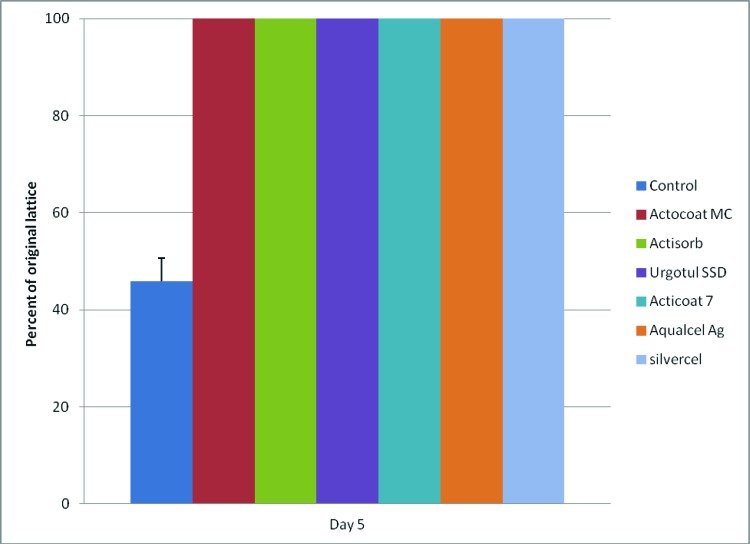
FPCLs did not contract because of lack of sufficient viable fibroblasts remaining after exposure to silver-containing dressings. Contreet Foam and Acticoat Absorbent absorbed all of the gel, thus preventing any data collection for those 2 dressings.

Contractions of the FPCLs were completely inhibited by all of the test silver-containing dressings compared with the control (*P* < .05) (Fig 1). At least 1 × 10^5^ viable fibroblasts are required in this model to produce lattice contraction. As can be seen in Table 1, Trypan Blue viability data demonstrated that an insufficient amount of viable fibroblasts remained to produce lattice contraction. This was corroborated by microscopy, which showed destruction of the fibroblasts. The FPCLs treated with Contreet Foam, and Acticoat Absorbent absorbed all the media in the gels thus no data were available for these 2 dressings.

**Figure 2:**
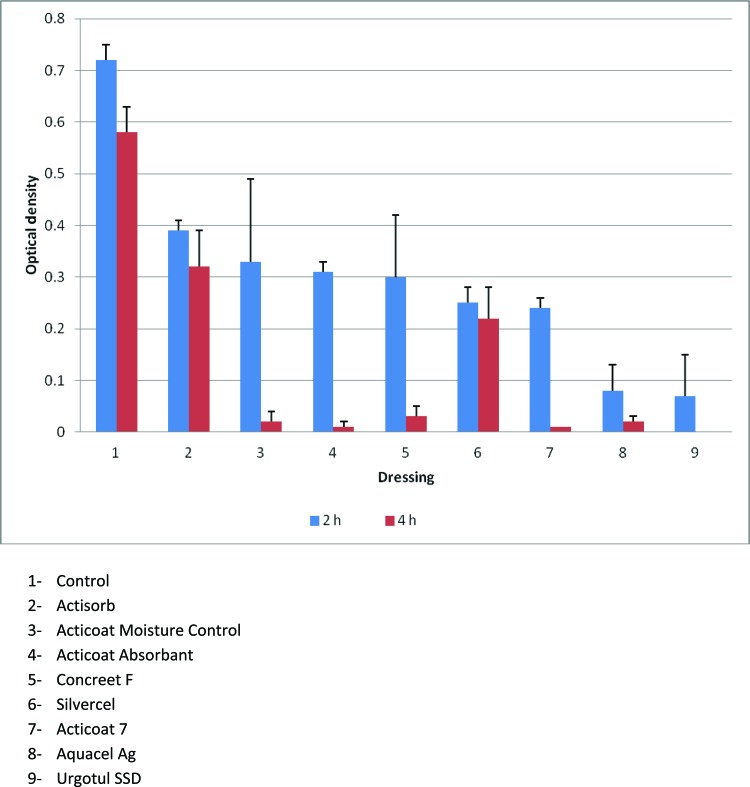
Results of the MTT Assay demonstrating markedly decreased mitochondrial activity in fibroblasts exposed to silver-containing dressings.

**Figure 3:**
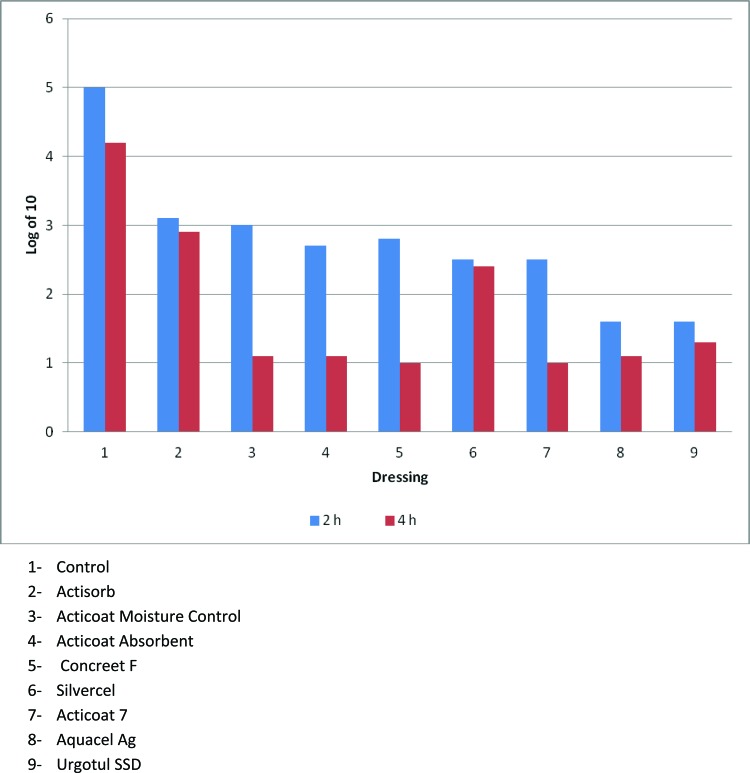
Conversion of mitochondrial activity to numbers of functioning fibroblasts shows that fibroblasts are markedly decreased after exposure to silver-containing dressings.

The mitochondrial testing allowed a true differentiation among the various dressings. Figure 2 demonstrates that Actisorb and Silvercel had significantly more mitochondrial activity at 4 hours compared with the other silver-containing dressings, but significantly less activity when compared with the control (*P* < .05). Figure 3 converts the optical density in Figure 2 to number of fibroblasts that expressed mitochondrial activity. Again, Actisorb and Silvercel were the least cytotoxic of the silver-containing dressings.

### In vivo comparative wound model

All of the silver-containing dressings decreased the tissue bacterial counts compared with the 2 control groups (Table 2). Contreet Foam did not eradicate all of the wound bacteria over the 10-day course of sampling, although it did decrease the CFUs per gram of tissue over 1000-fold. Five of the silver-containing dressings eradicated all wound bacteria within 48 hours (Table 2). The surface swabs identified the remaining bacteria in the wound as *E coli*.

There were marked differences among the dressings in their abilities to facilitate wound healing (Table 3). This model has historically required approximately 22 days to achieve complete wound closure. In the open control group (comparable to the historic control), 30% of the wound remained open at 12 days. Actisorb and Acticoat Absorbent Absorbent-treated wounds were essentially healed by day 12. All of the silver-containing dressings except Contreet Foam and Acticoat Moisture Control appeared to greatly accelerate healing of these contaminated wounds. Actisorb began to separate from the other dressings in its healing trajectory by day 8.

## DISCUSSION

Silver-containing solutions and compounds have enjoyed over a century of use as topical wound treatments.^13^ Incorporating silver into wound dressings is a more recent use of silver's antimicrobial properties. Silver is injurious to bacteria in several ways including damaging the bacterial cell wall and membrane permeability, blocking enzyme and transport systems, and preventing transcription and cell division.^17^ Since agents which are bacteriostatic and/or bactericidal are often also injurious to cells in the wound healing scheme, difficulties arise when attempting to evaluate the various silver-containing dressings and drugs as far as a benefit:risk ratio. Part of the difficulty has been due to use of only in vitro data to evaluate the antimicrobial or cytotoxic effects of various antiseptics and antimicrobials including silver. For instance, silver-containing dressings are often compared in the language of bacteriology.^13^ In vitro tests such as zones of inhibition, minimum inhibitory concentrations, minimum bactericidal concentrations, and bacterial log reductions are used to define an agent's antimicrobial effectiveness. This information is useful and allows comparisons of products. Similar in vitro tests are used to demonstrate potential cytotoxicity. Tests can demonstrate potential toxicity to keratinocytes, fibroblasts, and other cells in the wound healing scheme.^15^^,^^16^^,^^19^

The problem is that in vitro tests alone do not demonstrate the full efficacy, nor the potential harm of drug or dressing. In vivo tests often demonstrate a different picture. The combination of in vitro and in vivo test is useful in evaluating and comparing a benefit:risk ratio. The ultimate test may be the clinical trial, but for silver dressings the information is unclear. A 2010 Cochrane review of clinical trials on silver dressings concluded that there was insufficient evidence to determine the effectiveness of silver-containing dressings in promoting wound healing or preventing wound infection.^32^

Because the comparative antimicrobial effects of the various silver-containing dressings and drugs have been reported,^14^ the present study was performed to evaluate the possible injurious effects of these agents on wound healing. Although there are definite cytotoxic effects on the fibroblasts, as shown by the FPCL assay, the MTT assay, and the Trypan Blue assay, those effects do not appear to be severe enough to impede the remaining fibroblasts from allowing wound contraction. In fact, healing was accelerated with the use of most of the silver-containing dressings. The findings of in vitro fibroblast toxicity are similar to those reported in the literature.^15^^,^^16^^,^^19^ Not only has cytotoxicity been shown for normal fibroblasts, Zou et al reported cytotoxicity of silver dressings to human diabetic fibroblasts.^33^ In addition to fibroblasts, cytotoxicity to keratinocytes has also been reported.^15^^,^^16^^,^^19^ The effective killing of the tissue bacterial load seemed to be the best predictor of acceleration of healing. Actisorb, Acticoat Absorbent, and Urgotul SSD had the most rapid wound healing and all had effective in vivo antimicrobial results (Tables 2 and 3).

The lack of direct correlation of in vitro cytotoxicity and in vivo wound healing effects is not surprising. Kuhn et al^34^ explained that the FPCL was an in vitro method to evaluate fibroblast function and the effect that the fibroblast had at contracting a collagen lattice, and that for chronic wounds such as pressure ulcers, diabetic foot ulcers, and venous stasis ulcers, the FPCLs do not predict healing. Although silver sulfadiazine has been shown to have in vitro toxicity to keratinocytes, Bishop et al^35^ demonstrated that Silvadene (1% silver sulfadiazine, King Pharmaceuticals, Bristol, Tennessee) was more effective than the placebo in allowing epithelialization in a clinical trial of venous stasis ulcers. For that reason, a reproducible wound healing model is useful for evaluating wound healing effects. The contaminated wound model reported here demonstrated that silver-containing dressings appeared to benefit healing of the wounds. Similarly, Wright et al^36^ using a contaminated wound porcine model showed that silver-coated dressings improved wound healing by reducing wound levels of matrix metalloproteinases and increasing the level of apoptosis. The authors postulated that these effects of the silver would alter or compress the inflammatory events in the wound. Paddock et al^37^ also demonstrated an inhibitory effect on certain proinflammatory cytokines with the use of silver-containing dressings.

In conclusion, in vitro bacterial analyses do not fully predict the effect of an antimicrobial in the in vivo or clinical setting. The data presented demonstrate that in vitro cytotoxicity tests do not fully predict the effect of an agent on wound healing trajectories. Silver-containing dressings are not identical and affect both bacterial killing and wound healing with very different responses. Therefore, one must choose carefully when deciding on use of a specific silver-containing dressing or drug.

## Figures and Tables

**Table 1:** Fibroblast survival after 48 hours of exposure to silver-containing dressings

		Fibroblast Survival Count With
Dressing	pH	Trypan Blue Stain*
Control	7.05	361.5
Silvercel	6.68	26.8
Actisorb	7.41	16.0
Acticoat 7	6.80	3.4
Aquacel Abs	7.36	1.6
Urgotul SSD	6.90	1.2
Actisorb	6.85	0
Acticoat MC	6.93	No cells identified due to absorbency
Contreet Foam	7.09	No cells identified due to absorbency

*Average of 5 fields counted with a 10X microscopic lens.

Abs indicates absorbent; MC, moisture control; SSD, silver sulfadiazine.

**Table 2:** Tissue bacterial count in CFUs/gram of tissue*

Treatment	Day 2	Day 4	Day 6	Day 8	Day 10
Open control	2×10^5^	3.2×10^5^	4.6×10^5^	3.2×10^5^	3.2×10^5^
Closed control	1×10^5^	3.2×10^5^	1.2×10^5^	2×10^5^	1.2×10^5^
Actisorb	NG	NG	NG	NG	NG
Acticoat MC	NG	NG	NG	NG	NG
Urgotul SSD	NG	NG	NG	NG	NG
Aquacel Ag	NG	NG	NG	NG	NG
Acticoat Abs	NG	NG	NG	NG	NG
Silverlon	8×10^2^	NG	NG	NG	4×10^2^
Silvercel	2.5×10^2^	NG	NG	4×10^2^	NG
Acticoat 7	4×10^2^	3×10^2^	1.4×10^2^	NG	NG
Contreet foam	7×10^2^	8×10^2^	1×10^2^	7×10^2^	2×10^2^

*All wounds inoculated with 5×10^5^ CFUs on day 0.

Abs, indicates absorbent; CFU, colony forming unit; MC, moisture control; NG, no growth; SSD, silver sulfadiazine.

**Table 3:** Healing of acute contaminated wound model: percent of original wound remaining open

Treatment	Day 2	Day 4	Day 6	Day 8	Day 10	Day 12
Open control	95	83.5	77.5	65	47.5	*30*
Closed control	87.5	70	50	33.5	20	20
Actisorb	95	71.5	46.5	18.5*	1.5*	0*
Acticoat Abs	85	61.5	40	20*	10*	1.5*
Urgotul SSD	82.5	60	37.5	20*	8.6*	2.5*
Silverlon	72.5	48.5	32.5*	20*	10*	5*
Acticoat 7	75	53.5	32*	20*	11.5*	5*
Silvercel	72.5	51.5	35*	22.5*	15*	6.5*
Aquacel Ag	82.5	65	47.5	30	17.5	7.5*
Contreet Foam	91.5	77.5	60	42.5	23.5	15
Acticoat MC	97.5	85	67.5	48	25	15

*P < .05 compared with the closed control.

Abs indicates absorbent; MC, moisture control; SSD, silver sulfadiazine.
